# Quantum Simulation of the Silicene and Germanene for Sensing and Sequencing of DNA/RNA Nucleobases

**DOI:** 10.3390/bios11030059

**Published:** 2021-02-24

**Authors:** Hikmet Hakan Gürel, Bahadır Salmankurt

**Affiliations:** 1 Information Systems Engineering Department, Technology Faculty, Umuttepe Campus, Kocaeli University, Kocaeli 41001, Turkey; bahadir.salmankurt@kocaeli.edu.tr; 2 Department of Physics, Art and Science Faculty, Esentepe Campus, Sakarya University, Sakarya 54187, Turkey; 3 Remote Education Center, Sakarya University of Applied Sciences, Sakarya 54187, Turkey

**Keywords:** 2D material, silicene, germanene, DNA/RNA nucleobases, DFT, biosensor

## Abstract

Over the last decade, we have been witnessing the rise of two-dimensional (2D) materials. Several 2D materials with outstanding properties have been theoretically predicted and experimentally synthesized. 2D materials are good candidates for sensing and detecting various biomolecules because of their extraordinary properties, such as a high surface-to-volume ratio. Silicene and germanene are the monolayer honeycomb structures of silicon and germanium, respectively. Quantum simulations have been very effective in understanding the interaction mechanism of 2D materials and biomolecules and may play an important role in the development of effective and reliable biosensors. This article focuses on understanding the interaction of DNA/RNA nucleobases with silicene and germanane monolayers and obtaining the possibility of using silicene and germanane monolayers as a biosensor for DNA/RNA nucleobases’ sequencing using the first principle of Density Functional Theory (DFT) calculations with van der Waals (vdW) correction and nonequilibrium Green’s function method. Guanine (G), Cytosine (C), Adenine (A), Thymine (T), and Uracil (U) were examined as the analytes. The strength of adsorption between the DNA/RNA nucleobases and silicene and germanane is G > C > A > T > U. Moreover, our recent work on the investigation of Au- and Li-decorated silicene and germanane for detection of DNA/RNA nucleobases is presented. Our results show that it is possible to get remarkable changes in transmittance due to the adsorption of nucleobases, especially for G, A, and C. These results indicate that silicene and germanene are both good candidates for the applications in fast sequencing devices for DNA/RNA nucleobases. Additionally, our present results have the potential to give insight into experimental studies and can be valuable for advancements in biosensing and nanobiotechnology.

## 1. Introduction

Experimental synthesis of two-dimensional (2D) graphene [1] has led to the discovery of new 2D materials such as silicene [2], germanene [3], h-BN [4], MoS2 [5], stanine [6], and so forth. It is possible to form interfaces between 2D inorganic monolayers and biomolecules. These interfaces lead us to the discovery of several biomedical applications such as biosensors and smart drugs [7,8,9,10]. Advancements in nanotechnology allow us to detect the nanoscale events at the level of a single molecule by using 2D sensing platforms. DNA/RNA consists of molecules called nucleotides. Each nucleotide has a nitrogen base, a sugar group, and a phosphate group. The five types of nitrogen bases are Adenine (A), Guanine (G), Cytosine (C), Thymine (T), and Uracil (U). The properties of genetic codes are determined by the order of the nucleobases. Sensing and sequencing of DNA/RNA nucleobases are important in disease diagnosis, forensic science, and genomics [11]. Because of the extraordinary physical, mechanical, chemical, and tunable electronic properties, 2D materials have started to be preferred in the sensing of nucleobases. For instance, graphene, silicene, germanene, monolayer transition-metal dichalcogenides, MXenes, h-BN, and phosphorene are used for the detection of DNA/RNA nucleobases and amino acids [12,13,14,15,16,17,18,19,20,21,22,23,24,25,26,27,28,29].

**Figure 1:**
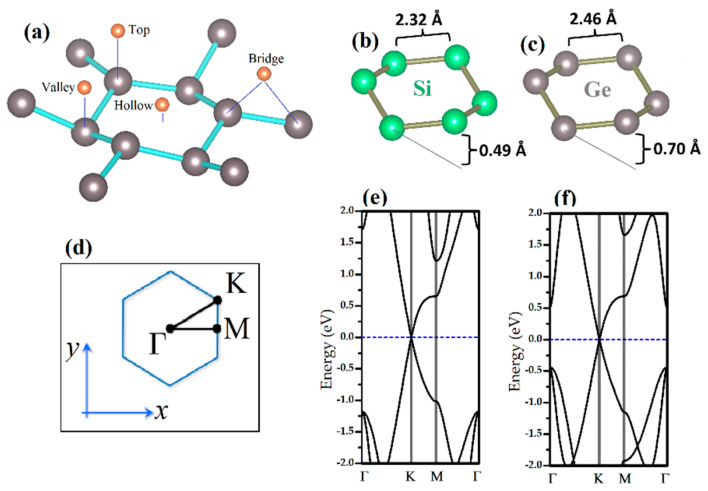
(**a**) Favorable adsorption sites: hollow, top, bridge, and valley for silicene and germanene. Buckled structure of (**b**) silicene and (**c**) germanene. Buckling and Si-Si and Ge-Ge distances are presented. (**d**) High-symmetry points of silicene and germanene. Band structure of bare (**e**) silicene and (**f**) germanene.

Over the recent years, graphene counterparts such as silicene and germanene have gained valuable attention in the scientific community. Silicene was successfully grown experimentally on Ag [2,30,31,32], Ir [33], and ZrB_2_ [34] substrate and germanene were also grown on the Pt (111) surface [35]. Although silicene and germanene stand out with their properties similar to graphene, they are different from graphene in terms of their structural properties. They have a buckled honeycomb crystal geometry (see Figure 1b,c), which provides for sp^3^ bonding. Because of their crystal structure, interaction of molecules and atoms with silicene and germanane monolayers is stronger than with graphene. This makes them potential candidates for the applications of sensing devices.

Despite the importance of the interactions of 2D materials with biomolecules, there are still limited studies with silicene and germanene. A covalent bonding mechanism between DNA/RNA nucleobases with silicene and germanene is absent in similar previous studies. Quantum simulations have been very effective in understanding the interaction mechanism of 2D materials and biomolecules and may play an important role in the development of effective and reliable biosensors. With the increase and widespread use of high-performance computing resources, it has become clear how powerful the first principle of the Density Functional Theory (DFT) is in modeling such systems. Metal-doped monolayers have better binding capabilities compared to pristine monolayers [17]. Gold atoms have some advantages over other metal atoms. The color of gold nanoparticles depends on their shape and size; they are highly preferred in nanomedicine.

In this work, we have calculated the binding of DNA/RNA nucleobases (NB) with pristine silicene/germanene and Au- and Li-decorated silicene/germanene by using the quantum simulations technique based on the Density Functional Theory (DFT), with van der Waals (vdW) correction and electronic transport properties, such as changes in the transmittance and conductance produced by adsorption of the nucleobases for the first time.

## 2. Materials and Methods

The interactions’ mechanism of nucleobases with the pristine and Au/Li-decorated silicene/germanene has been investigated using the Density Functional Theory (DFT)-based *SIESTA* package, where the basis set was used as a linear combination of numerical atomic orbitals (LCNAO) [36]. Electronic transport calculations were performed with nonequilibrium Green’s function (NEGF) formalism as implemented in the TranSiesta code [37]. The Perdew, Burke, and Ernzerhof functional within the generalized gradient approximation was used for the exchange–correlation functional [38]. The pseudopotentials that we used in this study are of the norm-conserving kind [39]. Conjugate gradient methods were used to optimize the geometry of the systems to get minimum energy configurations. Monkhorst–Pack meshes for Brillouin zone sampling were taken as 3 × 3 × 1 and 9 × 9 × 1 k-points for the relaxations and band structure calculations, respectively [40]. Furthermore, the Grimme-D2 method was used to describe long-range interactions [41]. For electronic transport calculations in the scattering region, the supercell had a size of 15.6 A^0^ × 20 A^0^ × 47.4 A^0^, while for the electrodes, we used a supercell with the size of 15.6 A^0^ × 20 A^0^ × 13.5 A^0^. A grid of 10 × 1 × 30 k-points was used for electronic transport calculations in the scatter region, while for the electrodes, the grid was composed of 60 × 1 × 30 k-points. The transport direction is aligned with the *z*-axis. The value of the mesh cut-off is taken as 300 Ry. We have employed periodic boundary conditions (PBC), so we have to give a space between two graphene layers in the adjacent supercells within 20 A^0^. Force tolerance was set to 0.04 eV/A^0^ for convergence. Due to the heavy metal Au atom, we have included a spin–orbit (SO) contribution to the calculations. Visualization of the systems has been performed by using *VESTA* software [42].

**Figure 2:**
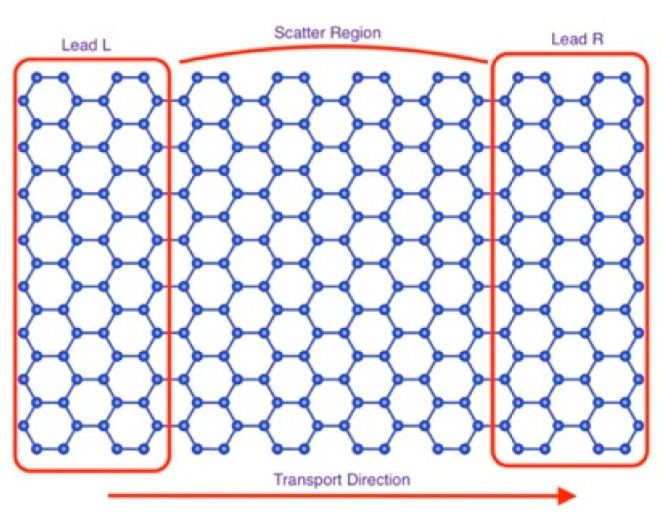
The sketch of the suggested silicene/germanene biosensor for DNA/RNA sequencing.

In the electronic transport calculation, the system was divided into three regions: two semi-infinite electrodes and one central scattering region. In our calculations, the electrodes were taken as pristine silicene for the right- and left-hand sides (see Figure 2). The scattering region consisted of the silicene layer and the DNA/RNA nucleobases.

The transmission can be obtained via the Green’s functions for this system

$$T\left(E\right)=\Gamma_{L}\left(E,V\right)G\left(E,V\right)\Gamma_{R}\left(E,V\right)G^{†}\left(E,V\right)$$

where the coupling matrices are given by $\Gamma_{\alpha }=i\left[\Sigma_{\alpha }-\Sigma_{\alpha }^{†}\right]$ with α≡{L,R}. $\Sigma_{L/R}$ are the self-energies of the semi-infinite electrodes and

$$G\left(E,V\right)={\left[E\;x\;S_{s}-H_{s}\left[\rho \right]-\Sigma_{L}\left(E,V\right)-\Sigma_{R}\left(E,V\right)\right]}^{-1}$$

where *S_S_* and *H_S_* are the overlap matrix and Hamiltonian, respectively, for the scatter region. The self-energies are given as $\Sigma_{\alpha }=V_{S\alpha }g_{\alpha }V_{\alpha S}$, where $g_{\alpha }$ are the surface Green’s functions for the semi-infinite leads and $V_{S\alpha }=V_{\alpha S}^{†}$ are the coupling matrix elements between the scattering region and the electrodes. The Hamiltonian H_S_ was obtained by the previous DFT calculations. Further information regarding NEGF can be found in [37,43].

## 3. Results and Discussion

Our recent results are detailed in the following sections. First of all, we investigated the binding mechanism of DNA/RNA nucleobases with bare silicene/germanene monolayers. By functionalizing Silicene/Germanene with Li and Au atoms, we have demonstrated that we can strongly binded Nuclebases to the functionalized surface. The obtained results indicate that Li and Au adatoms on silicene/germanene can significantly increase the binding energy of DNA/RNA nucleobases.

### 3.1. Binding Mechanism of Bare Silicene and Germanene Monolayers with Nucleobases

**Figure 3:**
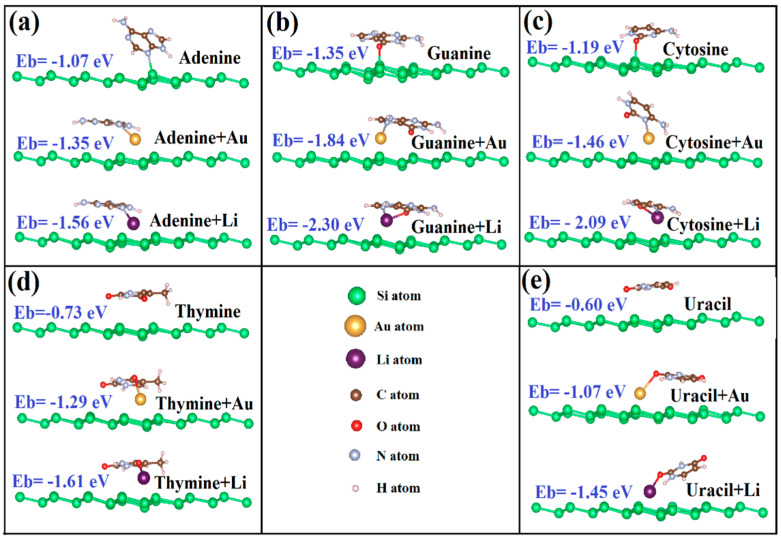
Optimized geometric structures and binding energies of nucleobases on bare and Li/Au-supported silicene. (**a**) Adenine, (**b**) Guanine, (**c**) Cytosine, (**d**) Thymine, and (**e**) Uracil.

**Figure 4:**
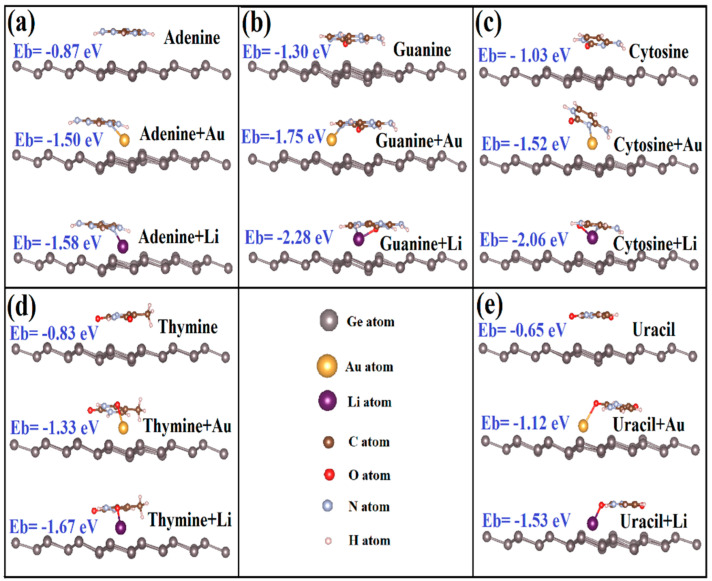
Optimized geometric structures and binding energies of nucleobases on bare and Li/Au-supported germanene. (**a**) Adenine, (**b**) Guanine, (**c**) Cytosine, (**d**) Thymine, and (**e**) Uracil.

The geometric relaxation covers both different absorption heights and orientations of DNA/RNA NB on the silicene and germanene. We examined the several possible orientations of NB on silicene and germanene layers; the most favorable structures of NB on silicene and germanene sheets are shown in Figure 3 and Figure 4, respectively.

It has been reported in earlier studies that being parallel is energetically more favorable because of the larger effective contact area for graphene [20]. According to our latest DFT calculations, all nucleobases have a tilted orientation on silicene. On the other hand, G, C, and T have a tilted orientation on germanene, but U and A show a parallel orientation. The tilting orientation provides sp^3^ bonding to strengthen chemical bonding between biomolecules and monolayers. We show that there is a chemisorption between G, C, A, and silicene. The oxygen atom of G and C and the nitrogen atom of A interact with the silicone atom of silicene, and they form a chemical bond. Our results are in agreement with the previous reported data except for A on silicene [14,20]. Amaorim et al. [14] and Hussain et al. [20] reported that A has a tilted orientation on silicene with no bonding. Our calculations predict bonding between the nitrogen atom of A and the silicone atom of silicene. However, there is no chemical bonding between nucleobases and germanene monolayers.

The binding energy, as shown in Table 1, can be defined, E_Binding_, as follows:

E_Binding_ = E_monolayer + NB_ − (E_monolayer_ + E_NB_) (1)

In terms of the total energies of the pristine silicene/germanene = monolayers (Li/Au-decorated, in some cases) and of the NB (NB = Adenine (A), Guanine (G), Cytosine (C), Thymine (T) and Uracil (U)), respectively. The binding energy can be taken as an indicator of the strength of the interaction between biomolecules and monolayers.

Our recent results show that adsorption energies are in the range from −0.58 eV to −1.35 eV for silicene and −0.65 eV to −1.30 eV for germanene. The order of adsorption energies of the NB with silicene/germanene is G > C > A > T > U. We can conclude from the results that the interaction of Guanine is stronger than the other nucleobases for both bare silicene and germanene. Uracil also has the lowest binding with bare silicene and germanene. The order of binding energies of nucleobases on silicene and germanene is consistent with the previously reported data [20]. In the case of silicene, Amaorim et al. [14] reported that C has the strongest binding among the nucleobases, and that the binding energy of A is equal to T.

The binding energy order is different from that of other 2D materials, such as graphene, MoS_2,_ and WS_2_ (G > A > T > C > U), calculated by the DFT-D2 method [18]. The difference possibly comes from the buckled honeycomb structure of silicene and germanene.

The closest adsorption distances between the monolayer atom/adatom and the closest pair of atoms between the silicene/germanene monolayers and nucleobases are shown in Table 2 and Table 3. Considering the most favorable adsorption geometries, in some cases, the reported distances correspond to bond length. There may be a correlation between binding energies and distances. More specifically, distances between Li/Au-supported germanene and the nucleobases are smaller than the distances between pristine germanene and the nucleobases.

### 3.2. Binding Mechanism of Li/Au-Doped Silicene and Germanene Monolayers with Nucleobases

According to our present results, in terms of binding energy, we confirm that the Li atom is chemisorbed onto the surface of silicene and germanene monolayers. The preferable adsorption site of the Au and Li atoms on silicene and germanene monolayers is a hollow site, see Figure 1a. Our calculated binding energy of the Li(Au) atom is −2.51 (−2.13) eV and −2.55 (−2.11) eV for silicene and germanene, respectively. Li and Au binding values are in good agreement with previous studies [44,45,46,47,48,49].

M. Sadeghi et al. [25] showed that the Li atom can improve the binding energy between the nucleobases and, MoS_2_. Song et al. [50] showed that the Au atom can increase the binding energy between the aromatic amino acids such as phenylalanine (Phe), tyrosine (Tyr), and tryptophan (Trp) on MoS_2_ monolayer. Additionally, Gorkan et al. [17] and Kadioglu et al. [51] reported that interactions of amino acids and nucleobases with Au clusters supported blue and black phosphorene, respectively.

In this part, we have calculated the binding of five nucleobases with the Li/Au adatom adsorbed to silicene and germanene monolayers. Li is an alkali metal and alkali metals are known as highly reactive metals. The equilibrium adsorption geometries with minimum total energies are shown in Figure 3 and Figure 4. The oxygen atom of G and C, the nitrogen atom of A, and one of the oxygen atoms of T and U interact with the Li atom on silicene/germanene, and they form a covalent bonding.

According to our recent calculations for Li (Au), the binding energies range from −1.45 (−1.01) eV to −2.30 (−1.84) eV and −1.53 (−1.12) eV to −2.28 (−1.75) eV for silicene and germanene, respectively. Although U has the lowest binding energy for both bare silicene and germanene, the binding energy of U increases 150% and 135% on Li-supported silicene and germanene, respectively. G has the strongest binding energy among the nucleobases; both bare and Li/Au-supported silicene and germanene.

This energy range is much more diverse than that achieved in pristine silicene and germanene monolayers. We say that the adsorbed Li and Au atom to pristine silicene/germanene monolayers can greatly increase the binding between the nucleobases and silicene/germanene monolayers. Li/Au + silicene/germanene monolayers preserve the order of binding energies with the NB, and it is G > C > A > T > U. This is easily seen in the optimized structure in Figure 3c,d, the Li + Silicene/Cytosine and Li + Silicene/Thymine, where the Li atom moves toward the bridge and valley site, respectively. Diffusion of the Li atom can be explained by Coulomb interaction between the Li atom and C/T. This is shown in the optimized structure in Figure 4b, the Au + Germanene/Guanine, where the Au atom diffuses to the next hollow site. Diffusion of the Au atom can be explained by the Coulomb interaction between the Au atom and G. Unlike Li on silicene/germanane, the nitrogen atom of G and C interacts with the Au atom on silicene/germanene, and they form covalent bonding.

### 3.3. Electronic Properties of Li/Au-Doped Silicene and Germanene Monolayers with Nucleobases

**Figure 5:**
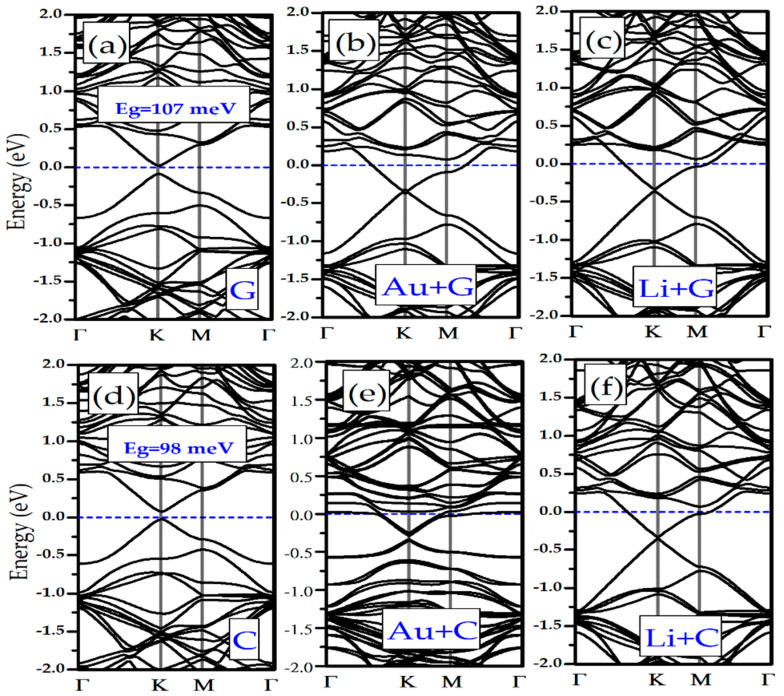
Electronic band structure of (**a**) Silicene/G, (**b**) Au + Silicene/G, (**c**) Li + Silicene/G, (**d**) Silicene/C, (**e**) Au + Silicene/C, and (**f**) Li + Silicene/C.

**Figure 6:**
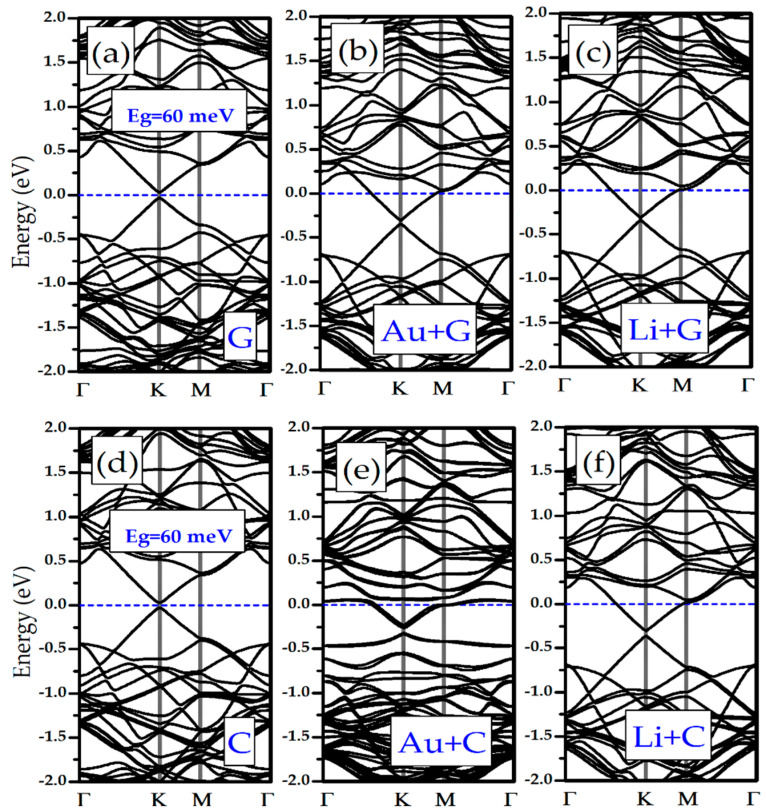
Electronic band structure of: (**a**) Germanene/G, (**b**) Au + Germanene/G, (**c**) Li + Germanene/G, (**d**) Germanene/C, (**e**) Au + Germanene/C, and (**f**) Li + Germanene/C.

We have investigated the effects of adsorption of Li/Au atoms in the electronic properties of silicene and germanene monolayers. In Figure 1e,f it is shown that pristine silicene and germanene are naturally semimetal and have a Dirac cone in K-point (see Figure 1d). Adsorption of the DNA/RNA nucleobases changes the band structure of bare silicene and germanene. In Figure 5a and Figure 6a,f, as examples of the DNA/RNA nucleobases, it is shown that there is a bandgap opening in Silicene + G, Silicene + C, Germanene + G, and Germanene + C. Adsorption of the DNA/RNA nucleobases on bare silicene and germanene causes a bandgap opening that ranges from 22 meV to 107 meV. We showed the maximum bandgap opening of silicene and germanene in Figure 5 and Figure 6, respectively. As a result of the adsorption of a Li and an Au atom, complexes of the semiconductor silicene and germanene/nucleobase complexes become metallic. This is due to the charge transfer from Li/Au atoms to silicene/germanene. Moreover, there is a small bandgap opening in the band below the Fermi level, and it occurs at the K symmetry point (see Figure 1d). For Au + Silicene/C see Figure 4e and for Au + Germanene/C see Figure 5e; there is a spin–orbit coupling that produces a band splitting.

### 3.4. Electronic Transport Properties

**Figure 7:**
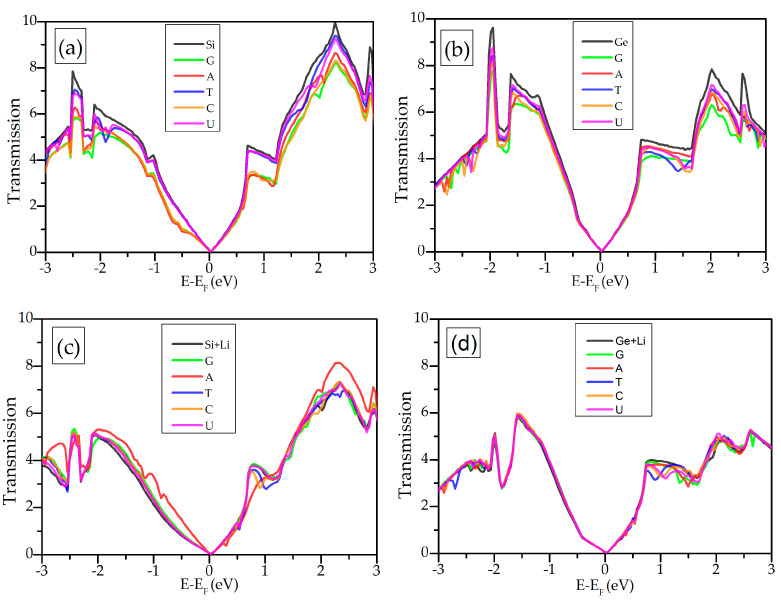
Transmission as a function of energy for each nucleobase on (**a**) silicene, (**b**) germanene, (**c**) Li-supported silicene, and (**d**) Li-supported germanene.

Here, we show the functionality of silicene and germanene for the DNA/RNA nucleobases’ sequencing. When the DNA/RNA nucleobases are adsorbed on the surface of the silicene, electronic transport properties of silicene/germanene will change. Transmittance T(E) shows the probability of an electron to be passed from one electrode to the other through the scattering region in the middle. In order to obtain electron transport properties, the transmission at zero bias voltage of the silicene/germanene attached to the semi-infinite electrodes is calculated, as shown in Figure 7a,d. Interaction of the DNA/RNA nucleobases with silicene/germanene monolayers causes reduction of energy-resolved transmission.

The main aim of this study is sensing and sequencing the DNA/RNA nucleobases by using silicene and germanene monolayers. We can design a theoretical biosensor for sensing the DNA/RNA nucleobases based on the variation in conductance. The sensitivity can be expressed as $S\left(\%\right)=\frac{\left|g-g_{0}\right|}{g_{0}}$, where g and $g_{0}$ are zero bias conductance of the biosensor with and without a nucleobase, respectively. For a small bias voltage, the conductance can be expressed as a function of gate voltage $V_{g}$, $g\left(V_{g}\right)=g_{0}T\left(E=\mu \right)$, where $g_{0}=\frac{2e^{2}}{h}$ is the quantum conductance, $\mu =E_{F}-eV_{g}$ is the chemical potential, h is the Planck’s constant, and e is the charge of the electron. The applied gate voltage ($V_{g}$) can tune the chemical potential to variation in the transmission. Measuring conductance rather than the current allows a better distinction of the change in the signal [52] because conductance through the biosensor changes by a unit of quantum conductance $g_{0}$ in its interaction with the DNA/RNA nucleobases.

**Figure 8:**
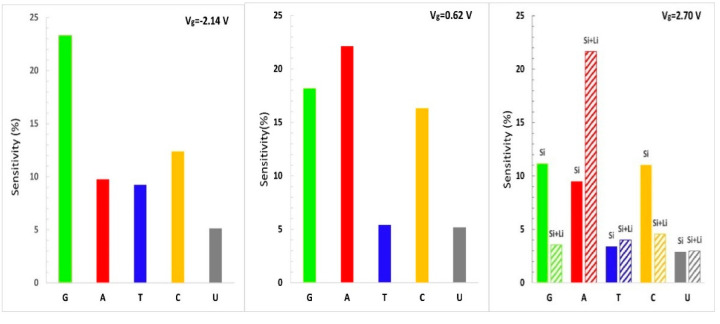
For different gate voltages (left panel V_g_ = −2.14 V and middle panel V_g_ = 0.62V), the calculated sensitivity histogram of the theoretical silicene sequencing biosensor with five different nucleobases is plotted. For the gate voltage V_g_ = 2.70 V (right panel), we compared the sensitivity histogram of the nucleobases on bare silicene (Si stands for silicene) and Li-supported silicene (Si + Li stands for Li-supported silicene).

**Figure 9:**
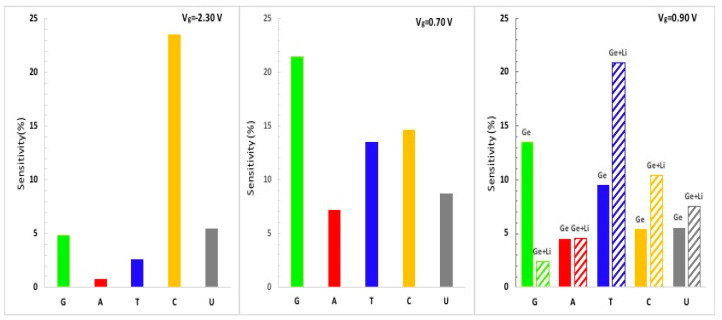
For different gate voltages (left panel V_g_ = −2.30 V and middle panel V_g_ = 0.70 V), the calculated sensitivity histogram of the theoretical germanene sequencing biosensor with five different nucleobases is plotted. For the gate voltage V_g_ = 0.90 V (right panel), we compared the sensitivity histogram of each nucleobase on bare germanene (Ge stands for bare germanene) and Li-supported germanene (Ge + Li stands for Li-supported germanene).

The sensitivity histogram of bare silicene/germanene (left and middle panel) and Li-supported silicene/germanene (right panel) is shown in Figure 8 and Figure 9, respectively. Our recent analysis shows that different conductance data from measurements at corresponding gate voltages allow distinguishing the nucleobases. At V_g_ = 0.62 V, it is possible to distinguish A from T and U. At V_g_ = −2.14 V, the theoretical silicene biosensor shows larger sensitivity for G and C and smaller sensitivity for U. At V_g_ = 2.70 V, and the bare silicene biosensor exhibits larger sensitivity for G, A, and C, while having smaller sensitivity for T and U. However, it is not possible to distinguish G, A, and C. A Li-supported silicene biosensor allows us to distinguish A from G and C for the same gate voltage. According to the sensitivity analysis of bare silicene distinguishing of T and U, it would not be as easy as G, C, and A. This problem can be solved by using a Li-supported silicene biosensor device. We note that the Li-supported silicene biosensor device can have be much more effective than the bare silicene biosensor device in distinguishing the DNA/RNA nucleobases.

According to the sensitivity histogram of the theoretical biosensor based on germanene, there is a larger sensitivity for G and C, as expected. Distinguishing T and U would not be as easy as G and C for the pristine germanane biosensor, but it is possible to distinguish T with Li-supported germanene.

## 4. Conclusions

In this manuscript, the adsorption behaviors of the DNA/RNA nucleobases on pristine silicene/germanane monolayer surfaces and Li/Au-supported silicene/germanene monolayers were investigated using DFT with vdW correction and the nonequilibrium Green’s function method. Our research covers all five DNA/RNA nucleobases in a nonaqueous environment. Our recent results show the sensing capability of pristine silicene/germanene and Li/Au-supported silicene/germanene and express their binding mechanisms. Au/Li atoms strongly interact with silicene and germanane monolayers. Silicene and germanene accept electrons from Au/Li adatoms, according to their greater electronegativity. The order of adsorption between the DNA/RNA nucleobases and silicene/germanane monolayers is G > C > A > T > U. The interaction of T and U with pristine silicene and germanene can be described as weak and noncovalent. T and U are physisorbed, while G, C, and A are chemisorbed on silicene. Adsorbents (Li/Au atoms) did not change the order of binding. It is clear that the adsorbed Li/Au atoms can significantly increase the binding between the nucleobases and silicene/germanene monolayers. The calculated orientations and bonding mechanism of the DNA/RNA nucleobases on silicene and germanene may play an important role for experimental studies to identify them by using scanning tunneling microscopy (STM). Adsorption of the DNA/RNA nucleobases on pristine silicene and germanene causes a bandgap opening at the K symmetry point. Li- and Au-supported silicene/germanene + nucleobases complexes become metallic. Additionally, in the case of C on Au-supported silicene/germanene, band splitting occurs below the Fermi level because of spin–orbit coupling. We also focused on the changes in electronic transport properties of bare and Li-supported silicene/germanane due to adsorption of the DNA/RNA nucleobases. We simulated that it is possible to distinguish G, C, and A from T and U due to changes in electronic signals. For the specific gate voltages, changes in transmission caused by the nucleobases differ from those of pristine and Li-supported silicene/germanene. Thus, it is possible to distinguish all the DNA/RNA nucleobases. Our findings show that both bare and metal-doped silicene and germanene are possible candidates for DNA/RNA sequencing biosensor applications.

Strong binding capacities of both bare and metal-doped silicene and germanene and tunable electronic properties of such systems indicate the possible applications of next-generation nanobiosensor technology for biomolecules. The obtained results will provide reference to both theoretical and experimental studies.

## Figures and Tables

**Table 1:** Binding energies of nucleobases on bare and Li/Au-supported silicene and germanene. Binding values are in eV. Negative binding energies indicate stable geometries.

Layer	G	A	T	C	U
Si	−1.35	−1.07	−0.73	−1.19	−0.58
Si [14]	−0.77	−0.60	−0.60	−0.88	
Si [20]	−1.16	−0.54	−0.70	−1.13	−0.43
Si + Au	−1.84	−1.35	−1.29	−1.46	−1.07
Si + Li	−2.30	−1.56	−1.61	−2.09	−1.45
Ge	−1.30	−0.87	−0.83	−1.03	−0.65
Ge [20]	−0.95	−0.70	−0.57	−0.56	−0.51
Ge + Au	−1.75	−1.50	−1.33	−1.52	−1.12
Ge + Li	−2.28	−1.58	−1.67	−2.06	−1.53

**Table 2:** The closest adsorption of distances between the monolayer atom (Si)/adatom (Li/Au) and the closest atom of the nucleobases. Values are in A^0^.

System	G	A	T	C	U
Si	(Si-O)	(Si-N)	(Si-O)	(Si-O)	(Si-O)
	1.95	2.02	2.77	1.92	3.28
Si [14]	(Si-O)	(Si-H)	(Si-H)	(Si-O)	
	1.99	3.07	3.07	2.04	
Si [21]	(Si-O)	(Si-H)	(Si-O)	(Si-O)	(Si-H)
	1.82	3.22	1.85	1.81	3.13
Si + Au	(Au-N)	(Au-N)	(Au-O)	(Au-N)	(Au-O)
	2.43	2.4	2.38	2.39	2.42
Si + Li	(Li-O)	(Li-N)	(Li-O)	(Li-O)	(Li-O)
	2.09	2.04	1.86	2.02	1.88

**Table 3:** The closest adsorption distances between the monolayer atom (Ge)/adatom (Li/Au) and the closest atom of the nucleobases. Values are in A^0^.

System	G	A	T	C	U
Ge	(Ge-O)	(Ge-H)	(Ge-O)	(Ge-N)	(Ge-O)
	2.41	3.17	2.96	2.61	3.09
Ge [21]	(Ge-O)	(Ge-H)	(Ge-H)	(Ge-O)	(Ge-O)
	2.26	3.05	3.02	3.09	3.12
Ge + Au	(Au-N)	(Au-N)	(Au-O)	(Au-N)	(Au-O)
	2.43	2.41	2.42	2.4	2.43
Ge + Li	(Li-O)	(Li-N)	(Li-O)	(Li-O)	(Li-O)
	2.12	2.06	1.9	2.03	1.9

## Data Availability

All data are contained within the article.
